# A weapon to fight against pervasive Omicron: systematic actions transiting to pre-COVID normal

**DOI:** 10.3389/fpubh.2023.1204275

**Published:** 2023-09-05

**Authors:** Na Wang, Jia Xue, Tianjiao Xu, Huijie Li, Bo Liu

**Affiliations:** ^1^School of Public Administration, Jilin University, Changchun, China; ^2^School of International Studies, Renmin University of China, Beijing, China; ^3^School of Literature and Law, Northeast Forestry University of China, Harbin, China

**Keywords:** COVID-19, pandemic control experience, risk management, systematic actions, Omicron, China

## Abstract

The Coronavirus Disease-2019 (COVID-19) pandemic is not just a health crisis but also a social crisis. Confronted with the resurgence of variants with massive infections, the triggered activities from personal needs may promote the spread, which should be considered in risk management. Meanwhile, it is important to ensure that the policy responses on citizen life to a lower level. In the face of Omicron mutations, we need to sum up the control experience accumulated, adapting strategies in the dynamic coevolution process while balancing life resumption and pandemic control, to meet challenges of future crises. We collected 46 cases occurring between 2021 and 2022, mainly from China, but also including five relevant cases from other countries around the world. Based on case studies, we combine micro-view individual needs/behaviors with macro-view management measures linking Maslow’s hierarchy of needs with the transmission chain of Omicron clusters. The proposed loophole chain could help identify both individual and management loopholes in the spread of the virus. The systematic actions that were taken have effectively combated these ubiquitous vulnerabilities at lower costs and lesser time. In the dynamic coevolution process, the Chinese government has made effective and more socially acceptable prevention policies while meeting the divergent needs of the entire society at the minimum costs. Systematic actions do help maintain the balance between individuals’ satisfaction and pandemic containment. This implies that risk management policies should reasonably consider individual needs and improve the cooperation of various stakeholders with targeted flexible measures, securing both public health and life resumption.

## 1. Introduction

To date, the number of confirmed cases of Coronavirus Disease-2019 (COVID-19) remains high worldwide. According to the WHO’s weekly epidemiological update on COVID-19, as of 13 November 2022, 632 million confirmed cases and 6.5 million deaths have been reported globally (1). Since it was first detected in South Africa in November 2021, Omicron has rapidly overtaken nearly all other circulating strains to dominate the globe. Of the more than 250,000 new Coronavirus sequences that have been recorded, 99.7% are Omicron strains, and they have evolved into numerous subtypes and recombinant viruses (2). It has been reported that the evolving Omicron variants are more transmissible, and their intergenerational transmission time is shorter (3, 4). Furthermore, Omicron cannot be easily detected using standard testing; hence, it is unofficially referred to as “Stealth Omicron” (5). These characteristics of Omicron have led to waves of faster outbreaks at a wider range, sabotaging previous efforts to combat COVID-19 (6). As explained by Tedros Adhanom Ghebreyesus, Director-General of the World Health Organization, the impact of COVID-19 pandemic on everyday lives has been immense. It affects all areas of life, such as economy, education, family, and employment (7). The various necessary activities of people may put them at a higher risk of infection and trigger COVID-19 clusters (8). Furthermore, the recurrence of the epidemic and the implementation of several preventive measures will cause anxiety and negative emotions among the public. To better cope with the uncertainty of the Omicron variant and the next waves of epidemic crises, it is necessary to address these issues and summarize the experience and lessons gained with the current epidemic management. With the constant mutation of Omicron, the relaxation of the prevention and control measures by the governments, the need to restore the economy, and people’s desire to return to their normal lives, measures to stop the spread of Omicron need to be improved further. WHO reported that the overall global risk related to Omicron in the context of the COVID-19 pandemic is still very high (9). As early as November 2021, WHO has alerted countries around the world to the Omicron variant, calling for solidarity, information sharing, and a coherent and consistent response plan on a global scale (10). In Spain, despite 82 percent of the population being fully vaccinated and half having had a booster shot, as of February 24,2022, the sixth wave of the epidemic caused by the Omicron have resulted in more than 12,000 deaths and more than five million confirmed cases, which equals more than all infections recorded during all other previous waves (4). On March 17, 2022, South Korea reported 621,328 new confirmed cases and 429 new deaths. This is the first time since the outbreak in South Korea that the number of newly confirmed cases in a single day exceeded 600,000, which also set a new single-day death record (11). In early March, infections rose sharply in Germany, with the national 7-day incidence rising from 1319.0 the previous day to 1388.5 infections per 100,000 people (12). As a country with a large population, China has faced many difficulties in fighting against COVID-19. For example, many families do not have homes suitable for home quarantine, and neighbors also have low acceptance of living near those who had contact with the virus. In addition, many industries could not implement the work-from-home setting for employees. The scarcity of health resources, especially in rural areas, also made it difficult to manage Omicron infection and posed increased risk of widespread outbreaks. Thus, government-led, multi-subject participation in epidemic management is necessary so that the epidemic could be contained. Under this circumstance, China has proposed to strive to achieve the maximum prevention and control effect at the least cost and minimize the impact of the epidemic on economic and social development the idea of “maximum prevention and control effect with minimum cost” (13). To achieve maximum prevention with minimum cost, China proposed the “dynamic zero-COVID” policy to eradicate the epidemic and prevent it from spreading locally. The essence of this measure is speed and precision (14). During the outbreak of the Omicron cluster infection in early 2022, China took a series of systematic actions to achieve the “dynamic zero-COVID” policy. These systematic measures combined with the high level of public cooperation significantly reduced the impact of Omicron and helped China survive the most difficult period. In the face of Omicron mutations, we need to sum up the control experience accumulated, adapting strategies in the dynamic coevolution process while balancing life resumption and pandemic control. This article focuses on China’s experience but also includes cases from other countries since the outbreak of Omicron. We show the process of virus transmission caused by different needs. The analysis integrates personal needs, personal behaviors, and management measures into the proposed loophole chain, which helps cascading effects to be intercepted more accurately and in a timely manner with lower costs, which explains the lower level of infections in China.

The aim of this article is to illustrate densely-populated countries, taking China as an example, can make effective and more socially acceptable prevention policies while meeting the divergent needs of the society at the minimum costs, even for the pervasive Omicron, that is, the system actions approach, before transiting to the new stage of post pandemic. The approach is by identifying individual and management loopholes in the spread of Omicron more precisely and quickly by the proposed loophole chain of our analysis, which can help fight against pervasive Omicron at lower cost and lesser time while also meeting the divergent needs of people. For densely-populated countries with scarce medical resources, systematic actions to fight against pervasive Omicron could be a cautious transition into next stage.

In addition to the combination of micro-personal needs and macro-governmental management, this paper has four major contributions to the analysis of pervasive Omicron cluster prevention. First, this article describes the complete process of cluster outbreaks in key groups and places, providing a direction toward future epidemic prevention. Second, it identifies tipping points, that is, personal and management loopholes, to help fight against pervasive Omicron with more precise systematic actions at lower cost and lesser time. Third, this article presents a detailed analysis of the high level of needs of individuals (self-esteem and self-actualization), which are being underestimated by the current research. The needs can ensure normal functioning of life and economy during the Omicron outbreaks in a positive way. This provides an important strategy for dealing with the Omicron crisis. Fourth, systematic actions provide a way to maintain the balance between individuals’ satisfaction and pandemic containment. This also implies that risk management policies should reasonably consider individual needs and improve the cooperation of various stakeholders with targeted flexible measures, securing both public health and life resumption.

## 2. Literature review

The epidemic has significantly affected people’s everyday lives. The virus also exhibited potential for mutation, which greatly threatens community health, social stability, and economic development. Countries have adopted different prevention and control measures to maintain people’s normal lives as much as possible. Aside from common viral testing, many other measures to prevent COVID-19 are also being applied, such as maintaining social distance (15), temporarily closing business (16), wearing face mask, getting vaccinated (17), prohibiting household mixing (18), restricting travel, and obeying stay-at-home orders (19). These practices have been proven effective. The lockdown policy in the United States has effectively reduced the COVID-19 death rate (20–23). Places where people often gather were severely restricted. For example, governments in more than 190 countries recommended temporary closure of schools during the outbreaks (24). However, there are still people who do not exactly understand the basic knowledge of prevention and have taken many wrong practices. For example, some parents let their children get infected with the virus in advance or some people avoid vaccination (25, 26). Although the WHO stated that the vaccine can effectively enhance immunity (27), many people still hesitate to vaccinate for the mislead information about the effectiveness of the vaccines (28). Furthermore, in promoting vaccination, the specific measures, support policies and strictness implemented around the world are different, which also brings about differences in vaccination rates. The differences in vaccination and effectiveness between countries will have a negative impact on the global prevention and control of Omicron (29). These situations all show that we need to explore a prevention and control model that incorporates past successful experience and adapts to the latest situation so as not to affect people’s normal life.

Although surveys in South Africa and other countries show that the mortality and severity of the fourth wave of infection have decreased (30), as the severity of the epidemic continues to increase, its prevention has become more difficult. The surge in Omicron cases is pushing many overburdened hospital systems to the edge, and healthcare systems are facing severe shortage of hospital staff (31). In addition, healthcare workers have become mentally, physically, and emotionally exhausted (32). Medical laboratories are also facing severe shortage of personnel (33). Omicron has had a “stacking” effect. The exacerbation of the patient’s condition is not only caused by the virus but also by the delays and interruptions in healthcare provision, resulting from overcrowding and understaffing in hospitals (34). Due to the increasing number of patients and shortage of medical staff, hospitals across the United States began suspending “elective” surgeries to ease the pressure on time (35). Meanwhile, human chronic infections is potentially contributing to further evolution and dispersal of the virus (36). The mixture of normal and special medical needs added obstacles to the normal allocation of hospitals. These real-world needs and historical issues combined with the new features of Omicron further increase the speed of infection (37). Coordinating the normal development of medical work and responding to the epidemic has become a challenge for the current prevention and control.

Some countries have also tried to make adjustments to maintain people’s daily needs. Since late December 2021, the number of countries affected by the epidemic has continued to increase (38), and the number of hospitalizations remains a challenge to the medical system (39). In the opinion of some researchers, unless public health policies are substantially changed, Omicron outbreaks in other countries are likely to occur with little to no warning (40). Due to the surge in the number of patients, many countries adjusted medical equipment according to the infection data (41). Countries such as Singapore, South Africa (41), and the United Kingdom (42) have made the adjustments according to the infection and hospitalization rates as well as the number of medical devices. The UK has also established an early warning tool, trying to extend the monitoring of medical staff to the society (43). In view of past successful experiences, some researchers suggested that hospitals formulate phased measures to ensure normal operation of public medical care (44). This type of alert system can provide dynamic practical guidance to respond to the evolving COVID-19 situation (45). Recognizing the high cost and insufficient resources due to the strict quarantine policies, many countries are working to develop models for predicting future outbreaks so as to rationally utilize medical resources (46). There are also researchers who monitor transmission and death through models to improve their predictive ability (47). Whether these monitoring and preventive measures are properly used has an important impact on the prevention and control results.

Aside from problems in medical treatment, other fields are also facing problems caused by the high prevention and control costs. To revive daily life and production needs, countries are looking for effective defensive measures with lower cost. Every country has its own regulations on the prevention and control standards as well as quarantine times and dynamically adjusts the degree of strictness. The strictness of the prevention and control policy is related to the country’s original institutional framework and governance philosophy. Countries in East Asia, such as South Korea and Japan, invested more resources in isolation and tracing and achieved remarkable results (48). In China, the cities relied more on vertical guidance, whereas New York assumed more coordination functions (49). However, most countries have great expectations on vaccine boosters. A study from the United Kingdom demonstrated that the viability of the virus significantly reduced after the third dose (50), and a research of Algeria also proved the importance of vaccines (51). Meanwhile, there have been new advances in vaccines against the original strain and the Omicron variant. The US Food and Drug Administration amended the emergency use authorizations of the vaccines Moderna and Pfizer–BioNTech to authorize bivalent formulations of the vaccines (52). This also proves that countries are interested in trying to explore low-cost prevention and control measures that are suitable for implementation in daily life.

However, the effectiveness of the previous policy has been weakened by Omicron. A bar in China still experienced cluster infections even after implementing temperature checks, disinfection, and crowd limits (53). Thailand, which adopted the etiquette of bowing rather than hand shaking, has also been hit by the new wave of the virus (54). The danger of Omicron is that existing vaccines are not fully protective against it; however, there are many unvaccinated populations who are at increased risk. Young people used to be hardly report to be infected but such cases are increasing (55). Studies have shown that measures such as wearing a mask, washing hands, social distancing and vaccination are still effective ways to fight against Omicron variant, and these measures cannot be gave up (56). Therefore, protective measures should be adapted to the actual situation and further improved, especially in key places such as schools, hospitals, and restaurants.

Under the pressure of reality, many countries had to adopt the strategy of dynamic adjustment. To balance economic development and medical health, some countries have made dynamic adjustments to the prevention and control measures. South Korea has eased restrictions. If the situation becomes stable, most social distance restrictions will likely be lifted (57). When Omicron first occurred, the United Kingdom tightened its prevention and control measures (58) but abandoned all anti-epidemic policies in February, for example, it lifted the mandatory self-isolation for people infected with COVID-19 (59), despite the occurrence of new Omicron variants (60). In the United States, the number of reported cases is also on the rise, with Philadelphia reinstating the requirement to wear masks indoors and reminding to protect oneself before the virus spreads (61). Meanwhile, China has proposed the “dynamic zero-COVID-19” policy (14) and started the fourth stage of comprehensive prevention and control with new management measures and forecasting models (62). Different regions within a country will need different levels of prevention and control policies due to the varying degrees of development (63), which also poses new challenges to the future epidemic prevention and control, that is, it is necessary to give each region some flexible adjustment space with the premise of consistent overall prevention and control objectives.

The large scale of the infected population as well as the fast infection transmission has caused greater social pressure than ever. Employees, especially in the service industry, are at a higher risk of exposure and are prone to burnout and nervousness. In December 2021, early in the Omicron outbreak, many retailers and restaurants in the United States were forced to close or shorten the drinking hours as there were not enough employees (64). The pandemic has also hit the food supply chain, posing greater risks to vulnerable groups (65). The case from the United Kingdom indicates that focusing on protecting vulnerable groups can effectively prevent the spread of the epidemic (66). Suppressing people’s daily needs is always the government’s last resort, but past experience has also given some ideas for normalized prevention and control. Cass Clay Food Partners, an American social network, had activated the power of community networks and quickly implemented a multipronged response to ensure the provision of people’s basic food needs (67). German civil society has strong potential to satisfy basic needs as it can provide mutual help and spontaneous respond to the outbreak, with young people helping the older adult to buy essentials in supermarkets and pharmacies (68). As the older adult in nursing homes are more vulnerable, the German government limited the movement of people in nursing homes and expanded the scope of nucleic acid testing for nursing home personnel (69).

Campuses are also key places that are prone to large-scale infections. American researchers have demonstrated that frequent testing coupled with strict attention to behavioral interventions (e.g., classroom infection control, scheduling and cohosting strategies, staff and teacher vaccination, and asymptomatic screening) is effective in preventing further COVID-19 transmission and outbreak in campuses (70). Groups such as medical workers, truck drivers, and catering operators are important in maintaining the normal functioning of society. However, they also need to protect themselves while shouldering the burden of household income. A study found that these groups are more likely to spread the virus within the family after work (71).

Some of the previous measures are still effective but are not enough to deal with Omicron and other possible variants that may occur later. Besides, long-term personal protection can cause behavioral fatigue (72). These subjective and objective reasons have made the current epidemic prevention and control more difficult, and many measures have obvious management loopholes that need to be further updated and improved. In addition, the media and public opinion will also affect the effectiveness of epidemic prevention and control policies. For example, inappropriate remarks will increase people’s resistance to vaccines and generate distrust in the event of public health emergencies, which are all important factors for the effectiveness of prevention and control (73). Individual activities are also closely related to the incidence of Omicron. Reducing outdoor activities and long-term isolation at home will bring many physiological and psychological problems, which is not conducive to the increase of individual immunity (74). Therefore, it is urgent to find a prevention and control model in daily life, and strengthen the effectiveness of previous policies and fill loopholes at the same time.

The loopholes are not only occurring in countries that lack infrastructure but also in countries with relatively complete protective chains. Despite Israel’s high level of development, children are also at risk of food shortages due to segregation. Furthermore, the low vaccination rates and a sudden increase in gatherings during the winter holidays have contributed to the effectiveness of the prevention and control policies (75). This type of situation also occurs in some underdeveloped areas where people are facing greater difficulties. In addition, the prevention and control in key places where people gather, such as bars, is not in place, and some places even provide ways to bypass restrictions (76). In view of this, this article attempts to explore the loopholes and countermeasures of the current prevention and control policies.

## 3. Case study: possible loopholes causing the chain of Omicron cluster infections integrating Maslow’s hierarchy of needs

Human needs are the basis of individual actions. Although the activities motivated by human needs may cause further spread of Omicron, satisfying human needs is the basic function of society. Some human needs are also helpful in containing the spread of Omicron and ensuring the normal functioning of society when the cluster epidemic spreads.

According to Maslow (77), human behaviors are motivated by five categories of needs from low to high levels. Physiological needs, such as the need for eating and clothing, are the most basic needs. Safety needs are related to security, order, and stability, including the risk of infection in the workplaces, homes, and the resulting disorder. Belonging needs refer to the need for love and is related to a person’s mental health. Since the start of the pandemic, there has been an increasing need for belonging among individuals. On the other hand, esteem needs include self-worth and respect from others, as well as recognition. Self-actualization includes needs for personal growth and self-fulfillment. In the face of the Omicron pandemic, self-esteem and self-actualization can make up for personal protection and management loopholes, thereby curbing cluster infections and maintaining normal functioning of society.

To present the context of human needs and activities that promote further transmission, we collected data and case details of Omicron cluster infection from official reports and authoritative media news. To provide a clearer picture of how personal and management loopholes evolve to an eventual cluster outbreak, we employed the case study approach (78) to provide deeper insights into the cluster outbreak in Omicron with detailed information about the cluster infection process, which may not be achieved when using other research methods.

Considering the information abundance for the chain analysis, the cases reported in the article are carefully selected to represent the correspondent Maslow’s scales. Among the five types of needs in Maslow’s hierarchy of needs, each has multiple forms, for example, physical needs include food, clothing, shopping, and transportation. With this circumstance and the fact that the characteristics of Omicron were not fully understood in the early stage, China withstood the pressure from different groups of people and different levels of needs during the Omicron outbreak in early 2022, reducing the impact of the virus on society and people.

When community cluster infection of Omicron has occurred, the virus has spread in multiple rounds in various ways, making it extremely difficult to find the clear transmission chain. However, the transmission chain was relatively clear before the community transmission, resulting in the clear discovery of the tipping point of cluster infection so as to intercept and contain the continued spread of the virus at the minimum cost. Therefore, community management and the cooperation of residents are both feasible and necessary for the prevention of the epidemic. This article uses figures and tables to clearly show the complete transmission chain of Omicron community infection in the case analysis. The figures present the possible loopholes causing the chain of Omicron cluster infections due to human needs, including loopholes in personal and management measures. Loopholes start from human needs, which cause people to be exposed to the virus and leading to its spread in public places with the appearance of crowds. The tables in the case study summarize the possible loopholes causing other cases of cluster infection due to related needs.

### 3.1. Case for physiological needs

**Figure 1:**
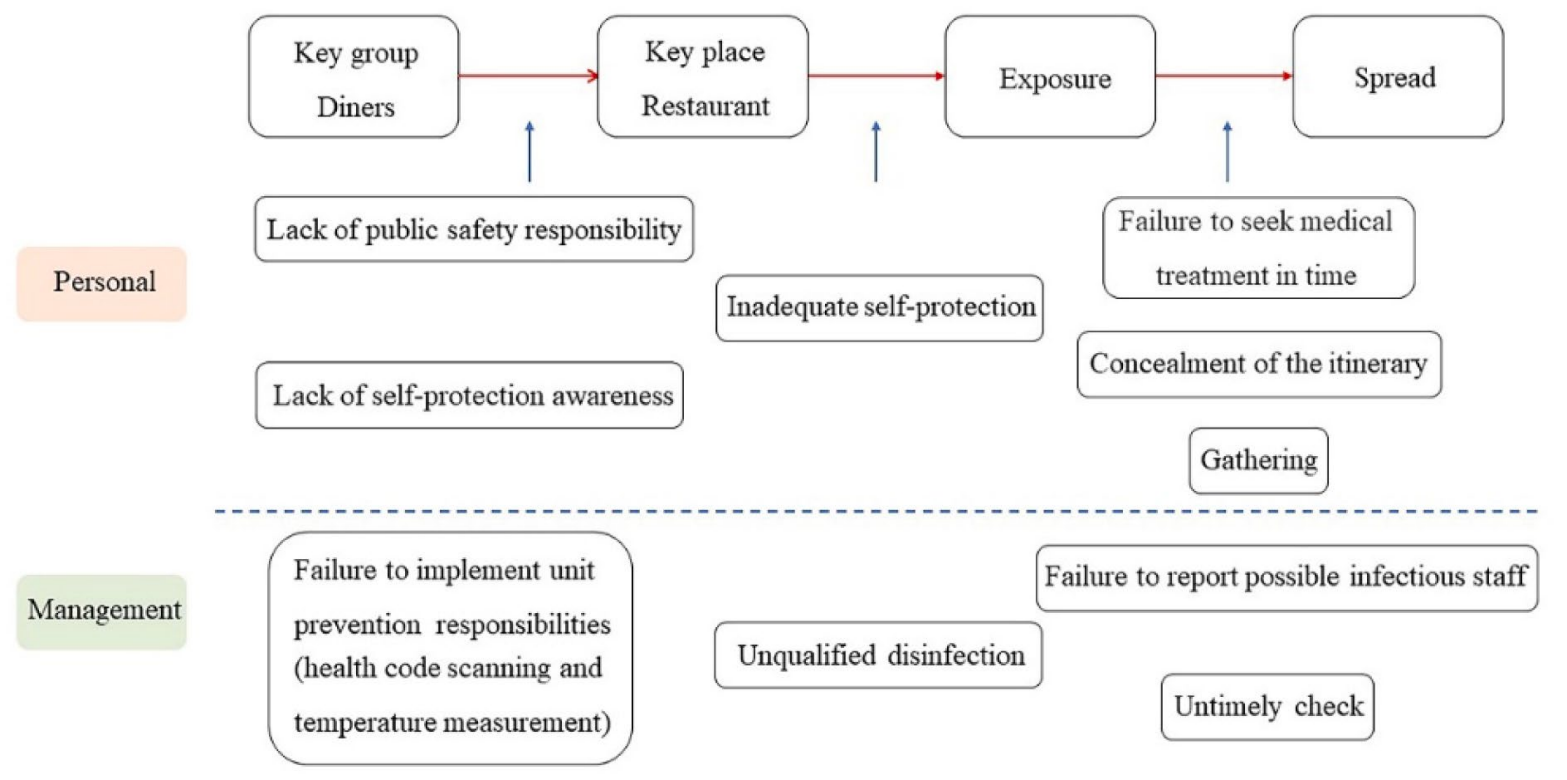
Chain of possible loopholes causing the chain of Omicron cluster infections among diners due to physiological needs.

Food, clothing, and transportation are basic needs for human survival. During the recovery period, both personal and management loopholes while meeting physiological needs led to the occurrence of Omicron infection cluster outbreaks. After the emergence of Omicron, cluster outbreaks frequently occurred in markets, buses or cars, clothing stores, etc. Based on the analysis of the collected clusters of infections (see Figure 1 and Appendix Table A1), the transmission of Omicron was caused by a series of personal and management loopholes while meeting physiological needs, such as lack of self-protection on indoor service, illegal operation, untimely health check, and unqualified disinfection.

In March 2022, a cluster infection occurred, which started in a restaurant in Beijing. As of March 22, the cluster has infected 17 people, including diners and their family members, as well as restaurant staff (79). Figure 1 presents the chain of possible loopholes that caused Omicron transmission due to food needs in the case of Beijing. The lack of public safety responsibility and the failure to seek medical treatment on time were the initial and most direct causes of this incident. The infected person (No. 50) had fever on March 7 but did not seek immediate medical treatment and instead went to the restaurant with five other people. The restaurant diners and staff did not take effective self-protection measures in a relatively closed space, which resulted in the spread of infection. The infected person, Liu, did not inform anyone of his dining experience and still wandered around despite the recommendation for home monitoring and nucleic acid testing. This posed a serious risk of epidemic spillover and increased the burden and difficulty of outbreak management.

Various management loopholes induced by restaurant owners are also key factors in this cluster infection. Although Beijing required all units to strictly implement customer health code scanning, many stores did not practice it. During the outbreak tracing, the disease control department found that the restaurant had not implemented health code scanning, temperature detection, health monitoring of employees, and other epidemic prevention measures. Furthermore, the restaurant operators did not report the condition of the temporary waiter, Zhang, which resulted in the exclusion of Zhang from the epidemiological investigation. This resulted in the spread of the epidemic, and 92 people in nine units, including hotels, restaurants, supermarkets, and wet markets, were monitored. To learn from this in combating Omicron, Beijing strengthened the implementation of health code scanning and posting of the warning sign “responsibility for epidemic prevention has not been implemented and there are high risks” outside the stores to warn customers.

Restaurants, supermarkets, and trains have relatively closed spaces and poor air flow. In these environments, personal and management loopholes could easily cause cluster infections. In November 2021, a cluster infection of Omicron occurred in Norway because of diners who spent too long in a relatively closed space and neglected self-protection (80). Customers and their families may be infected if the wet market does not strictly sterilize the food and its packaging, which was the case in Shenyang (81). Hiding the possibility of being infected and not cooperating with the management of relevant departments will also lead to the spread of infection. The confirmed case described here, namely, Zeng, tested positive in Shanghai on March 27 but refused to cooperate when he was subjected to a retesting; he also left Shanghai by train (82). This behavior is very likely to result in infection among travel companions and other urban residents. The fact that Zeng was still able to leave by train despite testing positive of the virus indicates the presence of loopholes in the management procedures and the poor information sharing between the different departments in Shanghai.

To balance the needs of traditional Chinese catering culture and epidemic prevention and control, the government hardly adopted large-scale policy measures to stop dine-in and to close shopping malls during the period of epidemic prevention and control. The checking of health code was only required upon entering closed, semi-closed, and crowded places. To facilitate nucleic acid testing for citizens, the government has improved the layout of standardized nucleic acid testing sites so that all residents can walk 15 min from their homes or offices to a free testing point. This would help improve their cooperation. Furthermore, some areas have explored the implementation of the meal-sharing method, strictly controlled the number of people gathering for meals, moved the dining venue to sparsely populated suburbs, and opened special takeaway windows. Henan, Gansu, and other places have combined dining restrictions with prohibitions on alcohol, which is stricter. At the same time, the society also calls on individuals to play a good supervisory role and cooperate with law enforcement to reduce the risk in dining areas.

### 3.2. Case for safety needs

After meeting the basic physiological needs, people will next seek security, hoping that the environment they live in is safe. The continuing epidemic and the emergence of Omicron variants have compromised people’s safe living space, from hospitals to other spaces, such as schools and nursing homes.

**Figure 2:**
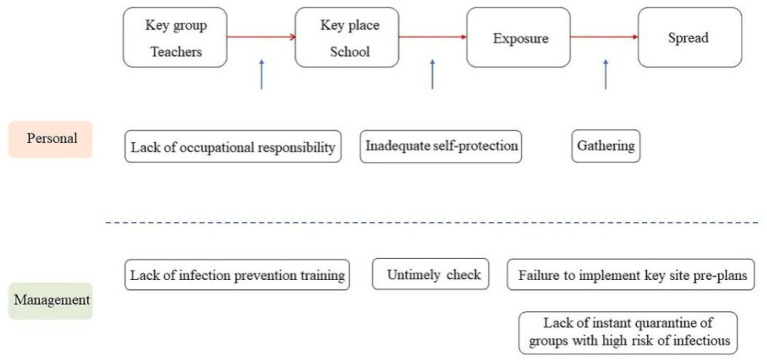
Chain of possible loopholes causing the chain of Omicron cluster infection in a teacher due to safety needs.

In March 2022, an intra-school cluster infection occurred in Jiutai District, Changchun City. Figure 2 presents the process of Omicron spread in this case. The source of the outbreak was a high school teacher who failed to comply with occupational requirements and still went to the school despite feeling unwell. The teacher’s personal loopholes included the lack of occupational responsibility as well as health monitoring and protective measures in school, resulting in the spread of Omicron during his gatherings with the students. The epidemic spread in Jiutai has become more insidious and faster than ever. Due to the shorter generation interval of Omicron, some people are contagious only within a day of being infected (83). In the described case, the campus management also had many loopholes and did not implement measures in accordance with “Technical Plan for the Prevention and Control of the New Coronary Pneumonia Epidemic in Primary and Secondary Schools” (84). There are omissions in the vocational education of schools, which leads to the lack of sufficient awareness of occupational safety among teachers. Furthermore, in the fore-mentioned case, the school did not conduct nucleic acid testing on returning teachers, which was supposedly required during the school season. Moreover, when the infection had occurred, the school did not subject the students and teachers to quarantine but only sent 5,000 students home, resulting in many close and subclose contacts and eventually leading to the large-scale community transmission.

**Figure 3:**
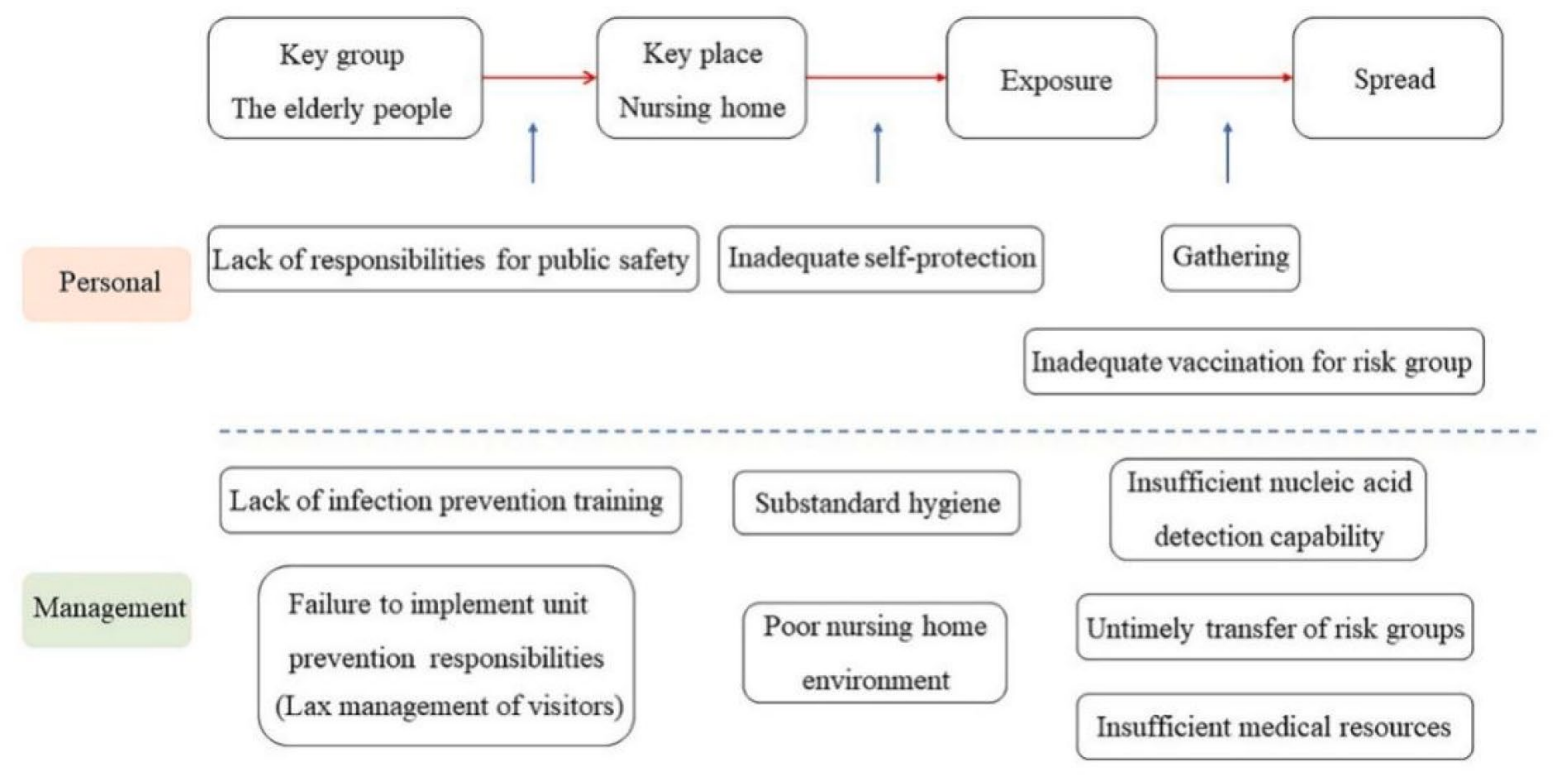
Chain of possible loopholes causing the Omicron cluster infections among the older adult due to safety needs.

Safe space encroachment has also occurred in older adult nursing homes in Hong Kong, China. A total of 360 nursing homes in Hong Kong have been attacked by Omicron, with more than 420 employees and 1,100 older adult infected (85). Figure 3 presents the whole process of this infection. From the figure, it can be clearly seen that the visitors entered the nursing homes without health check, indicating their lack of protection for the older adult. After entering the nursing home, they took off their masks and came into contact with the older adult. According to the data from the Centre for Health Protection of the Hong Kong Department of Health, the vaccination rate in nursing homes is only 34% (85). This is also one of the main loopholes causing the spread of the epidemic.

In terms of management loopholes, the Hong Kong government does not require staff below the level of nursing staff to undergo hygiene and infection control training, nor does it supervise the hygiene of nursing homes. Besides, due to the lax management, temporary workers in nursing homes often fail to perform disinfection in a standardized manner. In addition, nursing-home staff have a heavy workload and do not have sufficient time to care about the physical condition of the older adult. These have become potential dangers during the epidemic, and once Omicron occurs in nursing homes, it would be very difficult for them to quickly detect and effectively prevent the spread of the virus. Moreover, some nursing homes have stopped health screening on visitors, which enabled the infected people to enter the nursing homes. Also, the untimely transfer of groups with a high risk of infections also aggravated the spread of the epidemic.

The following are examples of the impact of Omicron on safety needs. Two schools in Howell, United States, had a cluster of Omicron infection in January 2022 (86). During the pandemic, many schools faced labor shortages as lots of teachers were unable to go to work due to the infection (87). Similarly, according to the self-reports of medical staff in the United States, the surge in the number of cases has also caused problems to them, such as fatigue and mental distress (88). As presented in Appendix Table A2, aside from schools, nursing homes, and hospitals, other spaces related to safety needs have also suffered from the encroachment of Omicron, such as quarantine hotels, logistics centers for sorting and express delivery, and wet markets.

There were also some positive cases that demonstrated that the elimination of personal and management loopholes in the virus transmission can contain the spread of Omicron. Compared with Jiutai teachers who lack occupational consciousness, the freight driver from Shanghai fulfilled his responsibilities very well. He underwent self-quarantine in the truck for 27 days to prevent risks to others (89). Jilin University adopted a positive management after Omicron emerged in the campus, switched to online learning, and implemented closed-loop management measures so as to stop the transmission of the infection in a timely manner (90).

In January 2022, the fifth wave of the outbreak in January this year was the most severe one ever seen in Hong Kong. This surge in the number of cases has resulted in a serious shortage of medical resources. Many patients needed to wait for treatment in tents or open spaces outside the hospital (91). Furthermore, the large-scale outbreak resulted in the failure to meet people’s security needs. Contrarily, Changchun, who experienced a large-scale outbreak at the beginning of the year, learned from the event, and although there were cases of infection in September and October 2022, the local government promptly established effective epidemic prevention policies, such as checking the negative nucleic acid testing certificate when entering closed, semi-closed, and crowded places; conducting nucleic acid testing for all employees in the city; and implementing centralized isolation of close contacts in a timely manner. The measures made the new cases all to be from a single personnel, and there were no cases of social infection, effectively preventing the spread of the epidemic. During the epidemic, the medical system of the city has operated normally, without stopping work and production, and has not disturbed the lives of the citizens, ensuring that their safety needs are met.

### 3.3. Case for belonging needs

**Figure 4:**
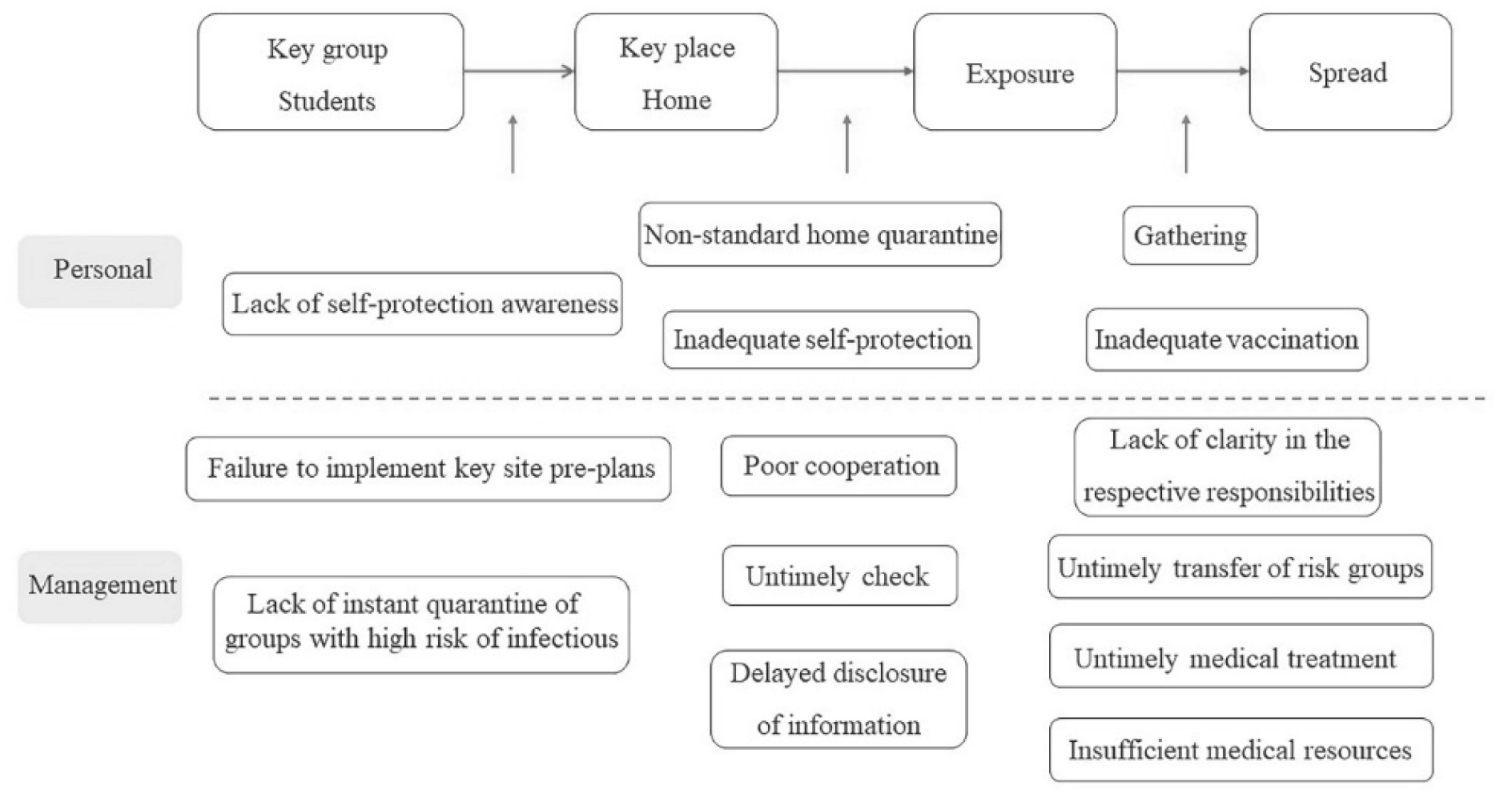
Chain of possible loopholes causing Omicron cluster infections among students due to belonging needs.

Belonging needs indicate that individuals desire to establish an emotional connection with others, the need for friends, and to seek solace in the group and family. Due to these needs, in the case of safety needs, we described the process of an intra-school cluster infection in Jiutai District, Jilin Province, but the cluster infection was not contained within the school. The school mistakenly sent 5,000 students’ home. These students, who experienced an infection at school, were in desperate need of emotional comfort from their families but then infected them when they went home. Figure 4 presents the loopholes in this community infection.

In terms of personal loopholes, the students lacked self-protection awareness and did not undergo health monitoring at home after coming into contact with the infected classmates and teachers. Their family members also did not protect themselves, thus resulting in their infection. There were also management loopholes in this case. The first one was sending home students who were possibly infected instead of subjecting them to quarantine. Furthermore, due to the failure to identify which department was responsible for the students and their parents, the nucleic acid testing was not immediately carried out. Meanwhile, for students who had undergone nucleic acid testing at school, their results were delayed, which led to cross-infection among the families of those who were positive. In addition, loopholes such as the lack of clarity in the respective responsibilities of the epidemic management and shifting of responsibilities have expanded the scope of Omicron spread. Moreover, Jiutai is far away from the urban area, and there are insufficient medical resources to deal with large-scale cluster infections, resulting in the untimely transmission of infections and lack of good treatment.

From groups with high risk of infections (students) receiving the school’s notification to return home, and to the large-scale spread in the community, multiple transmissions have been experienced during this period (see Figure 4). If the loopholes in this transmission chain are systematically managed, the spread of the epidemic could be prevented, and the number of infected people could be reduced effectively.

**Figure 5:**
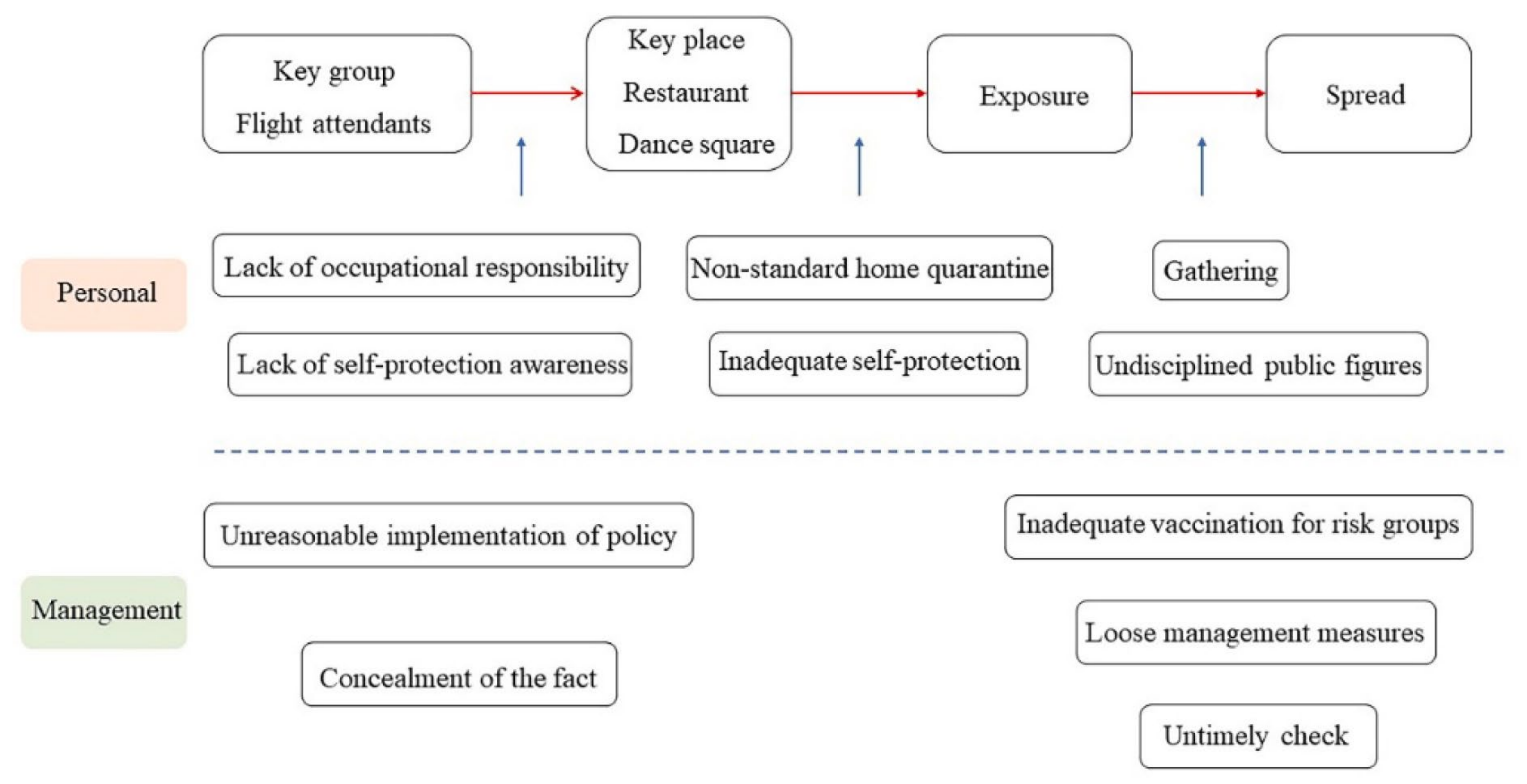
Chain of possible loopholes causing the Omicron cluster infection in a flight attendant due to belonging needs.

The community transmission in this case can be regarded as the secondary spread of intra-school infection based on personal emotional needs. The following case of Hong Kong is a daily life scene with a complete and independent transmission chain. A flight attendant returned to Hong Kong from Los Angeles on December 27, 2021, and tested positive for Omicron on December 31. During this time, the flight attendant stayed in the house, but her mother, who lived with her, was free to move. She danced at Victoria Park on December 31, with a scale of 20 people, causing the initial spread of the virus. Since then, some of the infected people have boarded the cruise ship, and some have participated in a concert on January 3, causing a wider spread. As of January 13, 2022, this cluster infection has spread for five generations, resulting in the infection of 24 people (92). Figure 5 presents the process of Omicron spread in this community cluster infection.

The Hong Kong government stipulated that passenger crews must undergo centralized quarantine after returning, whereas cargo crews can be exempted from this quarantine, that is, they only need health monitoring at home for 3 days. To avoid concentrated crew quarantine, Cathay Pacific took the liberty of converting all passenger aircraft operating on international routes to freighter status upon their return to Hong Kong (93). This action of the airline was a loophole that caused the virus to spread. Furthermore, the epidemic prevention department of Hong Kong did not carry out standard management of this flight attendant, which led to the unqualified home quarantine and eventually caused infection to the mother. As the mother was free to socialize to meet the need for belonging, she caused large-scale cluster infections in a crowded place due to the high viral load in her body because of the lack of vaccination (94). Another loophole in the management department was that the investigation was not timely and did not catch up with the spread of Omicron. When the epidemic in Hong Kong has spread intensively and the management department traced the source, it was found that the infected people went to a concert with nearly 10,000 people on January 3, 2022. This also indicates that the management and control departments failed to manage Omicron and had a lax attitude toward the epidemic. Hong Kong officials were also slack in protection. On January 3, 2022, hundreds of politicians violated regulations to participate in a birthday party, causing many people to be infected (95).

Appendix Table A3 presents other cases of cluster infections due to belonging needs. The characteristics of Omicron have resulted in many manifestations that have not been seen in the previous cluster transmission due to belonging needs. For example, the transmission activities become more and more routine, the transmission chain is longer, the speed is faster, and the number of infected people is increasing. In the face of daily reports of new confirmed cases, people’s inner pressure was increasing, and it was more necessary to relieve the pressure through social activities than usual.

There are also many positive examples of preventing the spread of the epidemic. For example, Mr. Lin, a 60-year-old man who was doing renovation work in Shanghai, was about to leave after finishing his work. However, he took the initiative to quarantine himself to help reduce the spread of the virus when the community implemented static management (96). By intervening in advance of groups with high risk of infections and regulating their behaviors that came from the belonging needs, we can have more time to fight the virus and the opportunity to interrupt community transmission with the least cost. Taking into account people’s needs for belonging, the government’s epidemic prevention policy has also made timely adjustments. Under the premise of scientific and accurate management and control of risk areas, the scope of control and personnel are minimized. At the same time, the isolation requirements are reduced, such as the 20 measures for epidemic prevention and control promulgated in November, 2022, which adjusted the “7-day centralized isolation” to “7-day home isolation” for spillover personnel in high-risk areas (97). According to the “Diagnosis and Treatment Plan for Novel Coronavirus Pneumonia (Trial Version 9)” issued by the National Health Commission of China, the nucleic acid CT value standard for discharge has been reduced, indicating that patients can be discharged early. At the same time, “continue isolation management and health monitoring for 14 days after discharge” was revised to “release isolation management or continue health monitoring at home for 7 days after discharge.” (98). Early discharge and isolation are possible, which meets the needs of quarantined people and isolated families for belonging. This not only allows infected people to return to their normal live as soon as possible but also avoids the waste of medical resources.

### 3.4. Cases for esteem needs

The epidemic has inspired the sense of responsibility and prompted people to cherish life by respecting each other and doing their best to contribute to society. Compared with the previous epidemic prevention and control, the current one includes not only ordinary citizens who make selfless dedication but also staff who stick to their posts. During the Omicron epidemic, more people participated in the prevention and control (see Table 1). These people have greatly safeguarded the basic functioning of the city during the lockdown and achieved respect for themselves at the risk of being infected. These cases also encouraged more people to spontaneously pass on love and responsibility by, e.g., collecting information to improve the mutual aid manual, and more volunteers to undertake coordination and guarantee work, which increased the vitality and warmth of the community.

**Table 1:** Contribution of different groups driven by esteem needs during Omicron cluster infections.

Identity	Duty	Functions	Reference number
Builders	Aid in the construction of the cabin hospital	Provide quarantine and medical facilities for infected people	(99)
Shopkeepers	Sell daily necessities	Provide basic items for daily necessities	(100)
Ride-hailing drivers	Transport passengers to their destination	Ensure the normal operation of urban transportation	(99)
Delivery men	Food and daily necessities material dispatch	Meet the food needs of the residents	(101)
Couriers	Receive and deliver couriers	Meet the shopping and living needs of the residents	(102)
Grassroots staff	Assist in outbreak management	Maintain the basic operation of urban and the needs of people’s lives	(103)

The strong transmission of Omicron has caused the number of infected people to remain high. Mobile cabin hospitals needed to be built to be used as quarantine places and for treating the infected people. In March 2022, after the Omicron outbreak in Changchun, Jilin Province, construction aid workers came from all over the country. The dedication of these workers ensured the rapid completion of the mobile cabin hospitals; however, the return to different regions and various loopholes in the process also led to the infection of many workers (104). Nearly 90 of the more than 160 workers from Harbin were infected (105). The same situation also occurred in Shanghai. In comparison, the whole process plan in Shanghai was more detailed and comprehensive, and various loopholes were blocked in time, and the transmission chain was cut off in the later stage (106). Certain risks existed in the construction assistance work, and adequate protection preparations were required in advance. However, due to the sudden and large-scale outbreak of the epidemic, the labor service company did not have sufficient time and plans to deal with the sudden event. In the early stage of Changchun mobile cabin hospital construction, there was a shortage of standardized management of personnel mobility; however, Shanghai had strengthened this management (107). Furthermore, construction workers from Shanghai arrived at the quarantine place *via* self-drive or company-chartered vehicles, achieving a closed-loop return. Among the 20 recent enhancements enacted in China, the level and number of quarantine days have been reduced, as well as the barriers to pursuing esteemed needs, and activities have been facilitated by the abolition of entry circuit breakers (97).

Nucleic acid testers, takeaways, grassroots government workers, taxi drivers, and ordinary citizens are also working hard to maintain the normal operation of the city during the cluster outbreak (108). To guarantee the supplies to surrounding residents during the citywide closure, from March 9 to 31, 2022, the manager of a convenience store in Minhang District of Shanghai, lived in the store without bedroom and shower for 23 days (99). For the aging villages with many older adult in need of a variety of medicines areas additionally, volunteers like Ms. Zhang stood out to be the grid leader for one village in Shanghai. She accurately identified the residents’ needs and connected with hospitals and pharmacies to help residents get medicines (100). During the raging period of Omicron, food supply was a major difficulty for the city due to the lockdown. To solve this problem, more than 3,000 JD.com couriers from all over the country voluntarily signed up to help Shanghai, and even some couriers arrived in Shanghai after 55 h of traffic. This action is called “suicide one-way express,” which fully demonstrates the responsibility and dedication of enterprises and individuals (109). In solving the “last 100 meters” delivery work, some young people took the initiative to become volunteers to undertake group purchases, disinfection, and delivery work and came up with innovative solutions to optimize the process (101). Furthermore, a 23-year-old girl posted a video of her volunteer work on the Internet, which encouraged more young people to do the same (102). At the same time, the government issued various measures to safeguard volunteer work (103). There were also some people who failed to respect themselves and others, causing harm to the health of others and the city’s epidemic management. For example, a man in Shanghai refused to cooperate after receiving the notification of nucleic acid retesting and went home by train, resulting in close contact with more than 200 people (110). A football player from Rome also failed to fulfill the responsibility of self-protection. He still attended his wife’s birthday party knowing that his wife tested positive for COVID-19 and then joined the team training (111). Individuals have a strong intention to meet the esteemed needs in their lives, which should be guaranteed to increase the overall well-being of society on the one hand and improve epidemic prevention and control on the other hand. When individuals awaken their sense of responsibility and intentionally realize their esteemed needs, they can maximize the cooperation with epidemic prevention policies and reduce prevention and control costs.

### 3.5. Case for self-actualization needs

The need for self-actualization has not subsided during the COVID-19 pandemic but has instead grown stronger under various epidemic management measures. During the Omicron epidemic, many examples of efforts to safeguard people’s self-actualization needs emerged. For example, parents and medical staff in mobile cabin hospitals created a good learning environment for children (112), and event organizers provided athletes with opportunities to break through and realize themselves (113). The Winter Olympics is a global sports event. Athletes from all over the world have trained hard for many years to show their best and look forward to realizing their self-actualization needs. In the face of the more cunning Omicron, the organizers of the Beijing Winter Olympics had taken various epidemic management measures to ensure the safety of the competition venue and smooth progress of the competition, thereby helping athletes achieve their goals. The successful holding of the Beijing Winter Olympics has provided experiences for other events to safeguard the self-actualization needs of individuals during the epidemic.

**Figure 6:**
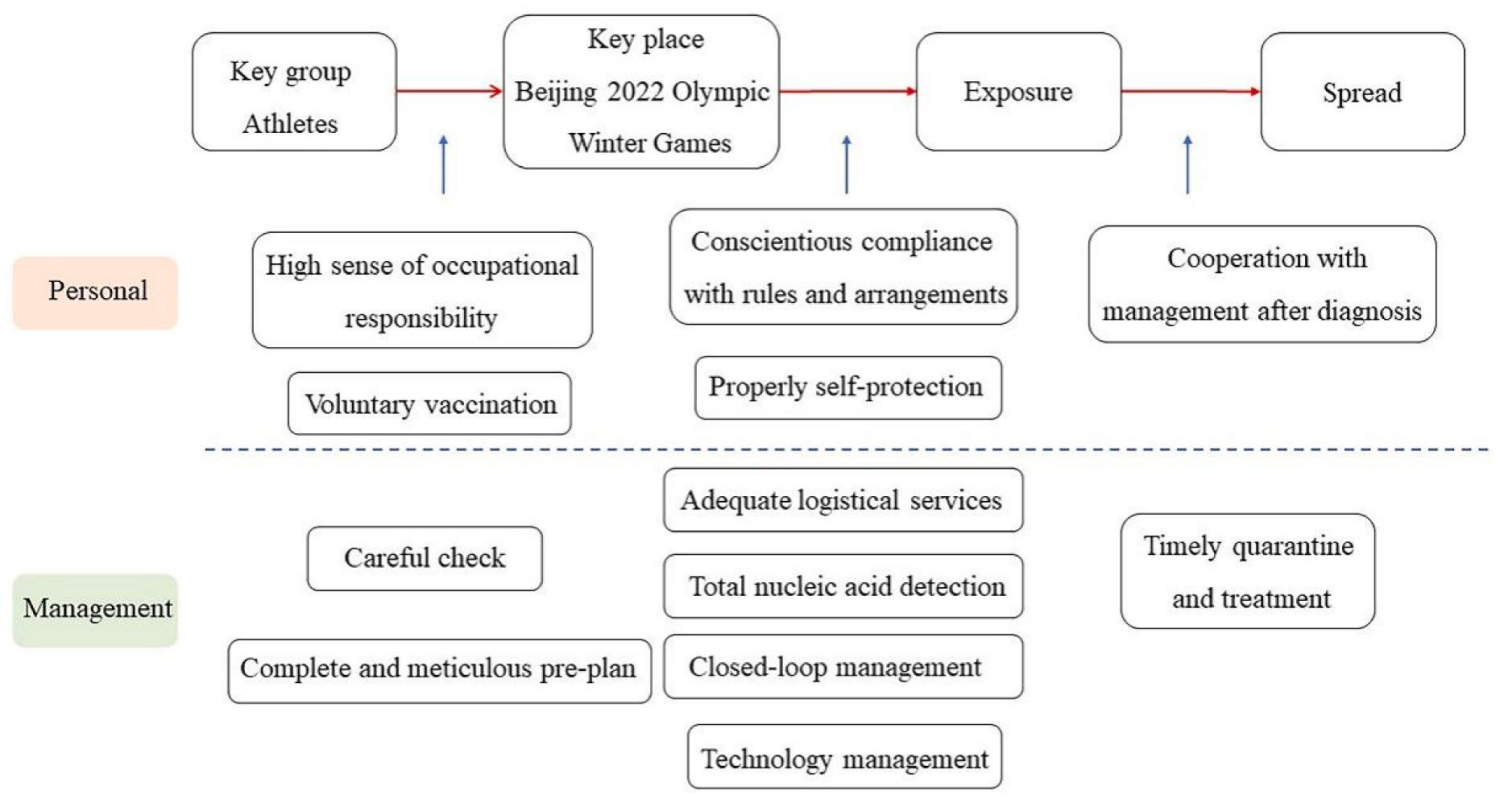
Chain of closing loopholes driven by self-actualization needs for athletes.

The success of this large-scale sports event is the result of efficient management measures, the cooperation of athletes, and the assistance of volunteers. The Beijing Winter Olympics has formulated detailed closed-loop management measures, covering all venues and links, such as arrival and departure, transportation, accommodation, catering, competitions, and opening and closing ceremonies (see Figure 6). Thomas Bach, president of the International Olympics Committee, said that the infection rate of COVID-19 in the Beijing Winter Olympics was 0.01%, and the “closed-loop system” was the safest epidemic prevention system (114). All the athletes completed the health checks before going to Beijing, and the management agency conducted a detailed health monitoring before the players entered the Olympic venues to prevent infected individuals from entering the closed-loop system. Athletes could only participate in training, competitions, and other activities in a specific space of the venue and use the special transportation system for the Winter Olympics. They stayed in their rooms as much as possible when not training or competing and wore masks in public places, such as the stadium. During the Beijing Winter Olympics, a total of 18 million nucleic acid testings were carried out, and each person was tested at least once a day (115). Athletes who test positive during the competition would be subjected to closed-loop quarantine. The 21-year-old American figure skating star Vincent Zhou tested positive in the second nucleic acid testing during the Olympics. He withdrew from the competition to secure the health of the others (116). Because the transmission chain was cut off on time, the epidemic did not spread. Figure 6 presents the various protective measures that the Winter Olympics administrators have arranged for athletes.

**Figure 7:**
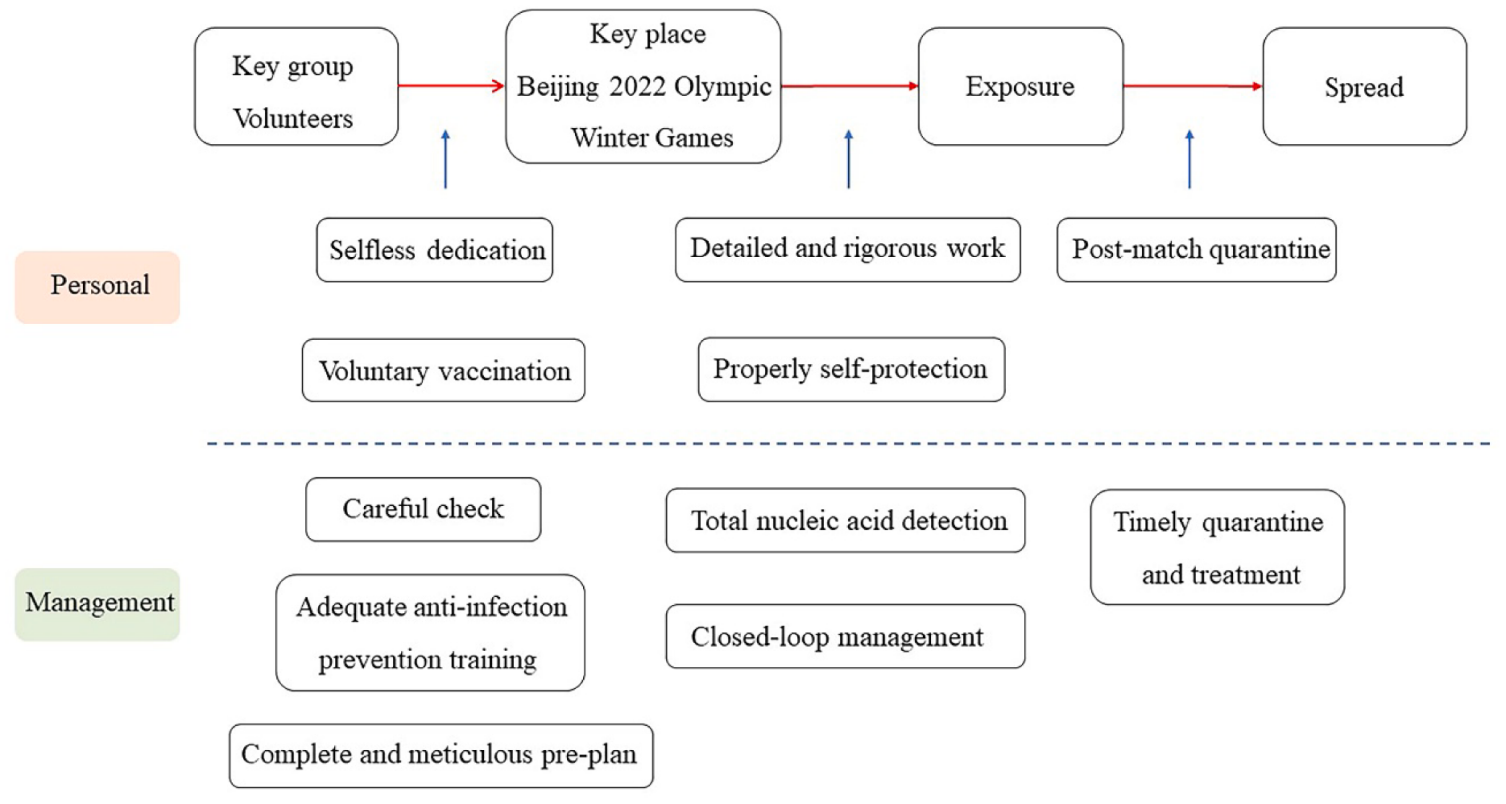
Chain of closing loopholes driven by self-actualization needs for volunteers.

The volunteers for the Beijing Winter Olympics were also very dedicated (see Figure 7). About 19,000 volunteers participated in the 2-month service work. During this period, they could not spend the most important festival in China, the Spring Festival, with their families. The medical staff, Ms. Liu, postponed her wedding ceremony to devote herself to volunteer work, and her husband applied to participate in the transportation guarantee work for the Winter Olympics (117). Volunteers received daily inspections and health protection education. They were instructed to follow the prescribed routes, which did not intersect or overlap with each other, and maintain a safe distance. Furthermore, they were subjected to a 21-day quarantine after the Olympics. After the Winter Olympics, some of the volunteers also devoted themselves to the epidemic prevention volunteer work in Hebei Province, which further enhanced their satisfaction of self-realization needs (118). Figure 7 presents the various protective measures that the Winter Olympics administrators have arranged for athletes.

While dealing with the epidemic, it is very important to meet people’s needs for self-realization and self-improvement. The scientific and humanized closed-loop management model of the Beijing Winter Olympics can provide experience for large-scale events, in which individual responsibility fulfillment, risk prevention and control, overall management, and rapid response are very important. High-tech products have also aid in completing tasks that lack manpower and provided more secure and sufficient guarantees. For example, robots can replace medical staff to achieve a 24-h uninterrupted disinfection. The 31st Summer Universiade, which will be held in Chengdu, China, in 2023, also draws on the closed-loop management model of the Beijing Winter Olympics and established the “one competition, one policy” epidemic prevention rules (119). During the epidemic, many self-realization activities were delayed or canceled, such as the Shanghai college entrance examination (120), indicating the importance of exploring the guarantees of realizing self-realization needs during the normalization of the epidemic. Many events that have been postponed in more than a dozen cities in China due to the epidemic are regrettable, such as the Gala Sports Awards and the Asian Cup, which have not met the higher demand of athletes and related personnel. In recent days, the WHO has called for the full use of major sporting events to encourage people to stay healthy and build stronger, more resilient health systems and communities (121). China has also recently introduced a more flexible prevention and control system, which included restrictions on lending and “point-to-point” transfer of important sports groups to face-to-face isolation closed-loop management areas, all of which provide convenient conditions to meet people’s needs for self-realization (97). As long as people are well prepared and have strong prevention and control, people’s self-realization needs are likely to be met, and a positive feedback can be generated to help control the spread of the epidemic.

## 4. Discussion

After the long and difficult period of the pandemic, many countries have adjusted health policies according to the features of Coronavirus variants (58–64). From March to May 2022, China also made a series of attempts and efforts under various uncertain circumstances (2). The experience of China in dealing with the loopholes in the massive cluster of outbreaks during this period and the subsequent recovery of the economy and public life can serve as a reference for other countries in similar situations. This article offers policy recommendations based on five cases, not only for the management departments but also for communities and individuals who have an important role to play, to achieve a more dynamic and effective prevention and control at the minimum cost.

This article aimed to find an effective and better weapon to fight against Omicron and its possible variants so as to shorten the processing time and reduce the scale of transmission and control. This weapon needs to be more precise in the positioning of groups at a high risk of infection, thereby reducing the affected groups and areas and ensuring normal life order and economic operation. By summarizing the experience in this fight of anti-epidemic process and analyzing the cases in the text that outbreaks of pervasive epidemics were generated to satisfy individual’s needs, we identified systematic actions to fight against Omicron. That is, in the dynamic coevolution process, the government has formulated effective and more socially acceptable prevention policies while meeting the divergent needs of the affected individuals, such as students, fresh markets, and enterprises. The cooperation of these individuals could normalize the entire social system at the minimum costs. Systematic action is based on sound policies established by the government, relies on social participation, and emphasizes community building and resident self-management. The multiple actors in the system action include governments, communities, epidemic prevention departments, key populations in the Omicron transmission chain, and people who live with or have been in contact with them, which we call key population contacts. System actions are specifically manifested in two aspects: first is the government’s formulation of effective and more socially acceptable prevention policies as well as provision of technical support and second, the mutual support and cooperation between various subjects in the system, through which the entire social system could return to normal at the minimum costs.

Systematic actions explain China’s effectiveness in the fight before the turn of COVID-19 variants into more transmissible. Since 2020, China’s epidemic prevention and control has undergone three stages: emergency containment of sudden epidemics, normalized prevention and control exploration, precise prevention and control of the whole transmission chain, and dynamic clearance. In response to the outbreak since March 2022, the Chinese government has learned from the experience in the process of fighting the epidemic, whereas in the process of dynamic coevolution, the government has formulated effective and more socially acceptable prevention policies while meeting the different needs of various groups, such as students and small businesses. The current prevention and control strategy is to stabilize and control the cluster epidemic and also to strictly prevent spillover and large-scale rebound. In view of the epidemic points, we will chase and block them one by one while strengthening the normalization of epidemic prevention and control.

Social participation has great advantages and can effectively solve the shortcomings of government management and limited resources. Extensive and orderly social participation can not only expand the intensity and breadth of epidemic prevention and control but also contribute to the integration of resources for epidemic management, as well as inspiring people and reducing panic. The adjustment of epidemic prevention policies relies on social participation, emphasizing community building and residents’ self-management. In the process of epidemic prevention and control normalization with systematic actions, it is necessary to protect the reasonable needs of citizens, minimize disturbances to citizens’ normal lives, and reduce citizens’ cooperation costs. Furthermore, it is important to strengthen the use of technical means to accurately delineate the scope of personnel who need to be isolated and avoid the use of large-scale centralized isolation, shutdown, and production prevention measures to balance the individual needs’ satisfaction and pandemic containment.

While improving epidemic prevention policies, the government also needs to pay more attention to special risk groups, mainly the older adult and children. They are at a higher risk of infection due to their low vaccination rates. In addition, older adults’ advanced age and multiple underlying diseases make them more susceptible and more severely infected. For this group, Changchun, Jilin Province, China, adopted nucleic acid testing at home for the older adult during the citywide lockdown. Every other day, the community would often learn about the needs of the older adult, deliver daily necessities such as vegetables and medicines to their homes every other day, and send vehicles to “point-to-point” medical treatment when medical need arises (122). This effectively protects the health and normal living needs of special risk groups during the epidemic.

The mutual support between the different subjects in the system are manifested in the cooperation between the key groups and the prevention and control departments; cooperation of close contacts with epidemic prevention work; and cooperation between the epidemic prevention departments. For the cooperation between key groups and the prevention and control departments, the first step is that individuals should maintain safety precautions and always stay alert to the epidemic. In all the negative cases in the article, the lack of self-protection awareness is present in every personal loophole. For people with high-risk jobs, such as teachers, flight attendants, couriers, truck drivers, etc., in addition to being aware of self-protection, they should also be occupational-conscious, strictly abide by the epidemic prevention regulations of related occupations, and accept infection control training. In April 2022, the Ministry of Education in China issued the “Guidelines for School Staff During Epidemic Prevention and Control (Trial),” which has been updated with the latest regulations on key populations, such as teachers and students, to adapt to the new epidemiological situation (123). Second, key groups also need to cooperate with the management departments in terms of behavior, e.g., by strictly implementing standard home quarantine. In the two cases of belonging needs, the key groups (students and flight attendant) did not abide by the quarantine policy and had come into contact with other groups, resulting in subsequent exposure and spread. The Centers for Disease Control and Prevention (CDC) in Beijing, China, issued the “Guidelines for Home Isolation and Observation during Novel Coronavirus Pneumonia Epidemic (4th edition)” in May 2022, based on the new features of the current virus transmission, to elucidate the criteria for home quarantine. Individuals should also be aware of self-protection by wearing masks, avoiding gatherings, and seeking immediate medical attention when feeling uncomfortable. This will reduce the exposure time when infected by Omicron and prevent the spread of the virus on a larger scale. Furthermore, not only does nucleic acid testing require individual cooperation, but the transfer and centralized quarantine of groups at a high risk of infection require even more individual understanding. People infected with Omicron are not easy to detect in the early stage, but they are already very contagious; thus, people who live or have a close contact with them are at a very high risk of infection. In this regard, communities are important in outbreak management. The community has the advantage of being in close contact with the public and can remind residents to pay attention to disinfection and protection during daily management. It can also provide care to the older adult and children. Vaccination is effective in preventing infection with the virus (124), and contacts of key groups should receive a booster vaccination. Due to the health code system in China, the close-contact group of confirmed cases can be identified through epidemic logical investigation. They should cooperate in the nucleic acid testing and transfer so as to quickly screen out infected individuals for treatment.

The systematic actions against Omicron also need to strengthen the cooperation between various departments, including operators and managers of key places, government management departments, and medical treatment systems. First and foremost, operators and managers of key places, such as restaurants and shopping malls, should comply with the relevant requirements issued by the epidemic prevention and control department and cooperate with their work. Although the situation is better now, there is no room for laxity, as many outbreaks have rebounded due to the negligence of managers. In the case of physiological needs, the CDC department of China found that the restaurant operators failed to implement epidemic prevention measures, such as health code scanning; temperature detection, which brought various problems and troubles to the subsequent epidemic source tracing; quarantine; and transfer of groups at a high risk of infectious. Second, the relevant government departments need to elucidate their respective responsibilities and strictly perform them. The negative cases of the paper all showed loopholes, such as untimely nucleic acid testing, unclear responsibility for joint prevention, and untimely transfer of groups at a high risk of infections by the government department. The government’s epidemic prevention department is the most important part in the entire anti-epidemic process. Only by ensuring its rapid and effective operation can it exert the cohesion and coordination of systematic measures to quickly stop the epidemic. Also, the medical system should fully cooperate with the overall epidemic prevention work. In view of the relatively weak medical capacity in some areas, medical treatment requires hierarchical diagnosis and treatment. In addition, the capacity of medical institutions needs to be strengthened by planning ahead to prepare designated and subdesignated hospitals, mobile cabin hospital, and central quarantine sites to ensure rapid activation in the outbreak (120). Hong Kong, China, proposes to allocate medical resources and strengthen the prevention and treatment of serious illnesses, as well as vaccination and effective quarantine in nursing homes, so as to remove risk points that are prone to serious outbreaks and deaths (125).

The systematic actions summarized in this article to fight against cluster infections are being flexibly adjusted according to the latest epidemic situation. After the success of the maskless World Cup with loosened policy responses against COVID-19, there have been 20 new measures by China’s in the fight transiting to the next stage, e.g., lowering the level of control, emphasizing precise prevention, and controlling and standardizing regional management policies. That is also the limitation of our study as polices are being dynamically adjusted considering the experience of other countries and the expectations of citizens. For example, various cities in China have hundreds of positive cases; however, mass testing for earlier identification have stopped. Residents who are homebound for a long time and do not need to go out may not take the nucleic acid test, thus avoiding the risk of infection due to aggregation. Nevertheless, systematic actions do prevent the spread of the virus on time and ensure the stability and order of people’s lives and economic resumption for quite a long period. For densely-populated countries with scarce medical resources, systematic actions as a cautious way contribute lower level of infections before transiting to next stage. This also implies that risk management policies should reasonably consider individual needs and enhance the cooperation of various stakeholders with targeted flexible measures, securing both public health and life resumption. Furthermore, the weapon of systematic actions can also help us maximize the effectiveness of epidemic prevention at minimal cost in future crises.

## 5. Conclusion

In view of the new challenges brought by Omicron’s characteristics, this paper summarizes some experiences of systematic actions to reduce the cost of epidemic prevention and the impact of various measures on people’s basic needs. Through cases study of different needs, we combine micro-action at the individual level with macro-management at the social level, using Maslow’s hierarchy of needs theory to analyze the epidemic spread chain. The personal and management loopholes identified can help prevent and control potential waves in the future. With the personal and management loopholes, the biggest challenge in preventing the spread of Omicron is speed and precision; otherwise, it may lead to the continued development of the virus transmission chain, triggering further community infection. The large-scale cluster epidemics in cases 1–3 were caused by the failure of the participants to carry out multiple links in the epidemic prevention system to actively perform their responsibilities, resulting in delays in prevention and control; thus, the epidemic had to be fought at the cost of wider control and more resource consumption. The latter two cases suggest that even in crowded situations, it is possible to keep people safe and meet their needs through dynamic management and efficient cooperation. This article offers policy recommendations based on five cases, not only for the management departments but also for communities and individuals who have an important role to play, to achieve a more dynamic and effective prevention and control at the minimum cost. Based on the China’s cases, we illustrated densely-populated countries made effective and more socially acceptable prevention policies while meeting the divergent needs of the society at the minimum costs with system actions. The mutual support and cooperation between various actors in the system, through which the entire social system could return to normal at lower costs and lesser time. Systematic actions have explained China’s effectiveness in the fight before COVID-19 variants turn into more transmissible.

The systematic actions summarized in this article to fight against cluster infections are being flexibly adjusted according to the newest epidemic situation. With the mutation of the virus, infection situations in the epidemic, the popularization of vaccination and the accumulation of domestic and foreign fighting experience, the prevention and control of the COVID-19 epidemic in China has entered a new phase. Under the premise of efficiently integrating the epidemic prevention and economic development, the management of COVID-19 in China was downgraded to a less stringent level. That is also the limitation of our study, because the polices are being dynamically adjusted considering the experience of other countries and expectations of citizens. For example, the targeting strategy for nucleic acid testing has been adjusted. Community residents will be tested when they want or need to be, and citywide nucleic acid screening is no longer conducted. The focus on personal protection is more advocated. Nevertheless, before this phase, systematic actions do prevent the virus spread on time and ensure the stability of people’s lives and economic resumption for quite a long period. For densely-populated countries with scarce medical resources, systematic actions may be difficult but a cautious approach with a lower level of infections fighting against pervasive Omicron before transiting to the next stage. This also implies that risk management policies should reasonably consider individual needs and enhance the cooperation of various stakeholders with targeted flexible measures, securing both public health and life resumption. Furthermore, the weapon of systematic actions can also help us maximize the effectiveness of epidemic prevention at minimal cost in future crises.

## Data availability statement

The original contributions presented in the study are included in the article/Supplementary material, further inquiries can be directed to the corresponding author.

## Author contributions

JX and NW: conceptualization, supervision, and project administration. JX, NW, and HL: methodology and formal analysis. JX, NW, and BL: resources. BL: data curation. JX, NW, HL, and TX: writing—original draft preparation. TX: writing—review and editing. NW: visualization. JX, NW, and HL: funding acquisition. All authors contributed to the article and approved the submitted version.

## Funding

This research was funded by the National Social Science Fund for Young Scholars, grant number 18CZZ010; the Integrity government construction research project of Jilin University, grant number 2018LZY003; the Social Science Fund for Young Scholars of Jilin Province, grant number 2021C18; the Fundamental Research Fund for Universities of Central Authorities, grant number 2572020BN01; the Fundamental Research Fund of Jilin University for Philosophy and Social Science Research Project, grant number 2017QY010; CPPCC theoretical research project of Jilin University, grant number 2021zx03002; and the Scientific Research Project of Jilin Provincial Education Department, grant numbers JJKH20220940SK and JJKH20231254SK.

## Conflict of interest

The authors declare that the research was conducted in the absence of any commercial or financial relationships that could be construed as a potential conflict of interest.

## Publisher’s note

All claims expressed in this article are solely those of the authors and do not necessarily represent those of their affiliated organizations, or those of the publisher, the editors and the reviewers. Any product that may be evaluated in this article, or claim that may be made by its manufacturer, is not guaranteed or endorsed by the publisher.
