# Supplementary material for: A weapon to fight against pervasive Omicron: systematic actions transiting to pre-COVID normal
Source: Front Public Health. 2023 Sep 5;11:1204275. doi: 10.3389/fpubh.2023.1204275 (PMC10512254; doi:10.3389/fpubh.2023.1204275)
Supplement: Supplementary file 1 [file Table_1.docx]

**Appendix Table A1. Possible loopholes in the chain of Omicron cluster infections caused by physiological needs**

| Key group | Key place | Date of first confirmed case | City | Cluster type | Personal loopholes | Management loopholes | Reference number |
| --- | --- | --- | --- | --- | --- | --- | --- |
| Diners | Restaurant | November 26, 2021 | Oslo, Norway | Catering | 1. Gathering   2. Unqualified personal protection | 1.Impossible complete segregation of staff and guests | 85 |
| Shoppers | Wet market | March 26, 2022 | Shanghai, China | Shopping | 1. Gathering  2. Unqualified personal protection  3. Failure to disinfect packaging after returning home | 1. Unqualified disinfection of public places  2. Loose control measures | 127 |
| Passengers | Train | March 31, 2022 | Wenzhou, China  Beijing, China | Travel | 1. Gathering  2. Unqualified personal protection  3. Unprotected prolonged eating in public places | 1. Unqualified disinfection of public places (Restroom door handle contaminated by a positive case)  2. Failure to screen the health of passengers | 128,129 |
| Passengers | Train | April 4, 2022 | Shanghai, China | Travel | 1. Failure to cooperate with retesting after a positive nucleic acid test  2. Conceal the fact of infection  3. Unqualified personal protection  4. Gathering | 1. Lack of close cooperation between prevention management departments  2. Untimely check  3. Loose control measures | 130 |
| Shop assistants and customers | Clothing store | March 29, 2022 | Dalian, China  Changshu, China  Beijing, China | Shopping | 1. Failure to disinfect packaging after receiving   2. Gathering  3. Unqualified personal protection | 1.Unqualified disinfection of imported products by import units  2. lack of clarity in the respective responsibilities | 131 |

| Vendors and customers | Convenience store | April 1, 2022 | Jilin, China | Shopping | 1. In-compliant operation  2. Lack of self-protection awareness  3. Unqualified personal protection  4. Unqualified disinfection  5. Gathering | 1. Lax supervision  2. Incomplete investigation | 132 |
| --- | --- | --- | --- | --- | --- | --- | --- |
