# Supplementary material for: A weapon to fight against pervasive Omicron: systematic actions transiting to pre-COVID normal
Source: Front Public Health. 2023 Sep 5;11:1204275. doi: 10.3389/fpubh.2023.1204275 (PMC10512254; doi:10.3389/fpubh.2023.1204275)
Supplement: Supplementary file 2 [file Table_2.docx]

**Appendix Table A2. Possible loopholes in the chain of Omicron cluster infections caused by safety needs**

| Key group | Key place | Date of first confirmed case | City | Cluster type | Personal loopholes | Management loopholes | Reference number |
| --- | --- | --- | --- | --- | --- | --- | --- |
| Drivers | Transport points | March 24, 2022  March 25, 2022 | Jinjiang, China  Suzhou, China  Nantong, China Yancheng, China  Dandong, China | Work | 1. Unqualified personal protection  2. Lack of occupational responsibility  3. Concealment of the itinerary | 1. Untimely check  2. Loose management measures | 133 |

| Cross-border truck drivers | Port | January 31, 2022 | Shenzhen, China | Work | 1. Lack of occupational responsibility  2. Unqualified personal protection | 1. Inappropriate policy  2. Insufficient disinfection of port  3. Untimely check  4. Loose management measures | 134 |
| --- | --- | --- | --- | --- | --- | --- | --- |
| Medical staff | Hospital | March 4, 2022 | Shanghai, China | Work | 1. Lack of occupational responsibility  2. Unqualified personal protection | 1. Untimely infection prevention training  2. Insufficient medical resources  3.Insufficient living equipment in centralized quarantine sites  4. Insufficient number of medical staff  5. Untimely transfer of groups with high risk of infectious  6. Poor cooperation between different departments  7. Inappropriate policy | 135 |
| Hotel staff | Hotels for quarantine | February 15, 2022 | Shenzhen, China | Work | 1. Lack of occupational responsibility  2. Unqualified personal protection | 1. Unqualified disinfection in hotel  2. Unqualified infection control training  3. Lax management over the quarantined personnel  4. Failure to implement the hotel's unit prevention and control responsibilities | 136 |
| Inbound passengers | Hotels for quarantine | April 6, 2022 | Shanghai, China | Quarantine | 1. Unqualified personal protection  2. Excessive flow of people  3. Gathering | 1. Mis-selected of quarantine location  2. Old hotel equipment  3. Unqualified disinfection  4. Untimely check | 137 |
| Shop owners | Wet Market | April 1, 2022 | Changchun, China | Work | 1. Lack of occupational responsibility  2. Unqualified personal protection  3. Gathering  4.Inadequate vaccination | 1. Unqualified disinfection in market including transport cars  2. Irregular food hygiene quality inspection  3. Failure to implement the market's unit prevention and control responsibilities  5. lax nucleic acid test | 138 |
| Courier | Courier storage point | April 12, 2022 | Taiyuan, China | Work | 1. Lack of occupational responsibility  2. Unqualified personal protection  3. Inadequate vaccination | 1. Untimely nucleic acid test  2. Unqualified disinfection  3. Failure to implement the courier companies' unit prevention and control responsibilities | 139 |
