# Supplementary material for: A weapon to fight against pervasive Omicron: systematic actions transiting to pre-COVID normal
Source: Front Public Health. 2023 Sep 5;11:1204275. doi: 10.3389/fpubh.2023.1204275 (PMC10512254; doi:10.3389/fpubh.2023.1204275)
Supplement: Supplementary file 3 [file Table_3.docx]

**Appendix Table A3. Possible loopholes in the chain of Omicron cluster infections caused by belonging needs**

| Key group | Key place | Date of first confirmed case | City | Cluster type | Personal loopholes | Management loopholes | Reference number |
| --- | --- | --- | --- | --- | --- | --- | --- |
| Wedding guests | Hotel | March 26, 2022 | Anyang, China | Wedding | 1. Lack of occupational responsibility  2. Lack of self-protection awareness  3. Unqualified personal protection  4. Gathering | 1. loose management measures  2. Failure to implement the hotels' unit prevention and control responsibilities | 140 |
| Customers | Restaurant | March 28, 2022 | Nanchang, China | Social | 1. Unqualified personal protection  2. Lack of self-protection awareness  3. Gathering | 1. Failure to implement the restaurants' unit prevention and control responsibilities  2. Untimely nucleic acid test  3. loose management measures | 141 |
| Customers | Bar | April 12, 2022 | Guangzhou, China | Social | 1. Gathering  2. Lack of self-protection awareness  3. Unqualified personal protection | 1.Indoor environment with poor air circulation  2. Untimely nucleic acid test  3. loose management measures | 142 |
| Members of Club | Concert | April 3, 2022 | Taibei,  China | Social | 1. Lack of self-protection awareness  2. Unqualified personal protection  3. Gathering | 1. Insufficient nucleic acid test  2. Untimely infection control training | 143 |
